# A New Index for the Quantitative Evaluation of Surgical Invasiveness Based on Perioperative Patients’ Behavior Patterns: Machine Learning Approach Using Triaxial Acceleration

**DOI:** 10.2196/50188

**Published:** 2023-11-14

**Authors:** Kozo Nakanishi, Hidenori Goto

**Affiliations:** 1 Department of General Thoracic Surgery National Hospital Organization Saitama Hospital Wako Saitama Japan

**Keywords:** surgery, invasiveness, triaxial acceleration, machine learning, human activity recognition, patient-oriented outcome, video-assisted thoracoscopic surgery, VATS, postoperative recovery, perioperative management, artificial intelligence, AI, mobile phone

## Abstract

**Background:**

The minimally invasive nature of thoracoscopic surgery is well recognized; however, the absence of a reliable evaluation method remains challenging. We hypothesized that the postoperative recovery speed is closely linked to surgical invasiveness, where recovery signifies the patient’s behavior transition back to their preoperative state during the perioperative period.

**Objective:**

This study aims to determine whether machine learning using triaxial acceleration data can effectively capture perioperative behavior changes and establish a quantitative index for quantifying variations in surgical invasiveness.

**Methods:**

We trained 7 distinct machine learning models using a publicly available human acceleration data set as supervised data. The 3 top-performing models were selected to predict patient actions, as determined by the Matthews correlation coefficient scores. Two patients who underwent different levels of invasive thoracoscopic surgery were selected as participants. Acceleration data were collected via chest sensors for 8 hours during the preoperative and postoperative hospitalization days. These data were categorized into 4 actions (walking, standing, sitting, and lying down) using the selected models. The actions predicted by the model with intermediate results were adopted as the actions of the participants. The daily appearance probability was calculated for each action. The 2 differences between 2 appearance probabilities (sitting vs standing and lying down vs walking) were calculated using 2 coordinates on the x- and y-axes. A 2D vector composed of coordinate values was defined as the index of behavior pattern (iBP) for the day. All daily iBPs were graphed, and the enclosed area and distance between points were calculated and compared between participants to assess the relationship between changes in the indices and invasiveness.

**Results:**

Patients 1 and 2 underwent lung lobectomy and incisional tumor biopsy, respectively. The selected predictive model was a light-gradient boosting model (mean Matthews correlation coefficient 0.98, SD 0.0027; accuracy: 0.98). The acceleration data yielded 548,466 points for patient 1 and 466,407 points for patient 2. The iBPs of patient 1 were [(0.32, 0.19), (–0.098, 0.46), (–0.15, 0.13), (–0.049, 0.22)] and those of patient 2 were [(0.55, 0.30), (0.77, 0.21), (0.60, 0.25), (0.61, 0.31)]. The enclosed areas were 0.077 and 0.0036 for patients 1 and 2, respectively. Notably, the distances for patient 1 were greater than those for patient 2 ({0.44, 0.46, 0.37, 0.26} vs {0.23, 0.0065, 0.059}; *P*=.03 [Mann-Whitney *U* test]).

**Conclusions:**

The selected machine learning model effectively predicted the actions of the surgical patients with high accuracy. The temporal distribution of action times revealed changes in behavior patterns during the perioperative phase. The proposed index may facilitate the recognition and visualization of perioperative changes in patients and differences in surgical invasiveness.

## Introduction

### Challenges in Defining Invasiveness

Contemporary surgery favors minimally invasive techniques, synonymous with endoscopic procedures, which are now widely used. However, robust evidence of their minimal invasiveness is lacking [1]. This may be attributed to the absence of a reliable method for evaluating surgical invasiveness.

Despite the advent of modern endoscopic surgery and its various indicators, the definition of invasiveness remains controversial. Parameters are mostly derived from medical perspectives, such as adverse event rates, analgesic use, and hospital stay duration [2-9]. Nevertheless, statistical differences in such indicators, unrelated to the patients’ daily lives, lack practicality and relevance [10].

### Focus and Objective of the Study

On the basis of our postoperative observations, the rapid behavior recovery following endoscopic surgery surprised us, particularly during the early perioperative phase. This observation led us to believe that minimal invasiveness is associated with the swift restoration of patients’ activity patterns.

Therefore, we aimed to establish an indicator that focuses on postoperative behavior recovery as a measure of surgical invasiveness. Patients gradually transition from rest to sitting, standing, walking, and finally returning to their preoperative lifestyles. A higher degree of invasiveness requires longer rest periods. This behavior change mirrors invasiveness. Knowing the behavior change as a quantitative indicator allows us to gauge invasiveness.

Although inpatient behavior patterns can be discerned by directly observing actions [11], human resources and privacy concerns arise, resulting in invasions of privacy. Therefore, we leveraged human activity recognition (HAR) technology to mitigate these issues by using machine learning and sensor data for activity detection [12-16].

Our investigation focused on the feasibility of understanding the behavior patterns of perioperative patients by using machine learning and a compact triaxial acceleration sensor. Moreover, we explored whether the knowledge of perioperative behavior patterns can yield a novel quantitative invasiveness index.

## Methods

### Ethical Considerations

This study was approved by the institutional ethics committee (R201519) and registered with the University Hospital Medical Information Network Individual Case Data Repository (UMIN000026843). We obtained written informed consent from all patients.

### Patients

#### Overview

Among patients undergoing thoracoscopic surgery for lung lobectomy or tumor biopsy for thoracic malignancy at our institute (National Hospital Organization Saitama Hospital), 2 patients who could walk independently before surgery and who provided written consent to participate in this study were included. One patient was admitted to a 36 m^2^ inpatient room with a toilet shared by 4 patients. The patient ate food from a bedside table and chair.

Perioperative medical care was provided in accordance with institutional clinical pathways. The patient was allowed to walk from the morning of postoperative day (POD) 1 with no obligatory transfers from the hospital room, except for daily chest radiographs. No behavior restrictions were imposed on the patient, and no rehabilitation was performed. Epidural analgesia was administered to control postoperative pain.

The following factors affecting the perioperative course were recorded: background (sex, age, and medical history); surgical procedure; operative time; blood loss; date of their walking resumption; drain removal date; amount of analgesics used; and duration of postoperative hospitalization.

A wearable sensor (myBeat; UNION-TOOL Corporation) 40.8×37.0×8.9 mm in size was used to measure accelerations, including gravity, in 3 orthogonal axes. The sensor was affixed to the center of the anterior chest of each participant. Data were measured from 9 AM to 5 PM on the preoperative day and during the postoperative period from POD 1 to the day before discharge.

Computer programs for data processing, analysis, and statistical testing were implemented using Python (version 3.9.16).

#### Selecting Learning Models to Predict Patients’ Actions Among Classifiers

Seven classifier models were trained and evaluated: decision tree, logistic regression, linear-type support vector machine, kernel-type support vector machine, random forest (RF), gradient boosting classifier (GBC), and light-gradient boosting method (LGBM).

The classification models were trained using supervised data and the k-fold cross-validation method and then compared using the Matthews correlation coefficient (MCC) and accuracy as evaluation indices. A grid search was performed to determine optimal parameters. The features were validated using the correlation coefficient and importance scores among the decision tree–type estimators.

The 3 top-performing learned models with the highest MCC percentages were selected as the learning models to predict the patient’s actions.

An open data set from the University of California School of Information and Computer Science repository was used as supervised data for machine learning. The data set was created at the Universitat Politècnica de Cataluña (UPC). Six actions were labeled in the UPC data set: WALKING, WALKING_UPSTAIRS, WALKING_DOWNSTAIRS, SITTING, STANDING, and LAYING. The data set contained 941,056 training data points. The raw acceleration training data from the data set were used as the supervised data set.

#### Data Preprocessing

Acceleration data were obtained as 3D vector data for the x-, y-, and z-axes. The axis directions were set to the x-axis of the data for gravity, y-axis for the patient’s right side, and z-axis for the patient’s rear side.

The measured data were separated into body acceleration vectors (BodyAcc) and gravity acceleration vectors (GravAcc) using bandpass filters. The BodyAcc magnitude was calculated as the root sum of the squares of BodyAcc. The Euler angles were calculated using GravAcc: the pitch and roll angles of the body axis in the direction of gravity.

The primary data set for calculating the features comprised 3 BodyAcc components, 3 GravAcc components, the BodyAcc magnitude, and 2 Euler angles.

The added features of the frequency component calculated from the metrics in the primary data set with fast Fourier transformation and 6 features for machine learning were used as test data: the median and SD of the acceleration value, frequency with the maximum amplitude, maximum amplitude value, phase, and mean frequency.

The primary data set and features for the training data were created from the raw data in the UPC data set using the same process as that used for the test data.

#### Prediction Aggregation

The actions of the patients were predicted using the 3 learning models selected. The actions predicted by the model that showed an intermediate prediction among the 3 models were considered as the actions of the patient.

In addition, the daily frequency of each activity was calculated. By dividing the frequency by the measured time of day, the proportion of time each action appeared per unit of time was calculated and defined as the action-specific indicator of the day, representing the appearance probability (AP). The appearance time (min) of an action per hour was calculated by multiplying the AP by 60.

To prevent accidents, patients were not allowed to use the stairs at the facility. Therefore, by combining 2 actions (WALKING_UPSTAIRS and _DOWNSTAIRS) with the WALKING action, 6 action classification labels were recounted into 4 categories: walking, standing, sitting, and lying. The 4 APs—walking (APwk), standing (APst), sitting (APsi), and lying down (APly)—were summed to 1.

### Calculating Behavior Pattern Indices

A chart with x- and y-axes was prepared, and 4 vectors with 4 orthogonal directions were placed on the chart. The 4 vectors were APly at coordinates (0, APly); vAPwk (0, –APwk); vAPsi (APsi, 0); and vAPst (–APst, 0).

The index of behavior pattern (iBP) was created from the 4 AP vectors (*vAP*) to easily recognize the day-by-day transition of the AP balance in the chart. The new index was defined as the center-of-gravity vector coordinates of the 4 *vAP*. The iBP has a vector with coordinates (x, y) using the following formula:

*iBP*(x, y) = ((APsi – APst) / 2, (APly – APwk) / 2) **(1)**

The *iBP* on each measurement day was plotted on a chart to evaluate the participants’ iBP changes.

The following additional indicators were calculated to evaluate the iBP:

1. The area enclosed by the line segments sequentially connects the coordinates of the daily
  iBP.
2. Day-by-day distance between 2 sequential points of
  iBP
  from day-n to day-n+1.

|*iBP_n+1_* – *iBP_n_*| (n=0, preoperatively)

1. Distance from the starting point: between the iBP point on day n and the preoperative day.

|*iBP_n_* – *iBP_0_*| (n=0, preoperatively)

1. Total sum of the day-by-day distances from the starting point.

∑|*iBP_n_ – iBP_0_*|

## Results

### Patients

Table 1 presents the participants’ characteristics. Patient 1 was a man aged 70 years who underwent thoracoscopic resection of the right lower lobe of the lung for lung cancer. Patient 2 was a man aged 71 years who had undergone thoracoscopic biopsy of an enlarged mediastinal mass (malignant lymphoma).

**Table 1:** Characteristics of the patients.

				Patient 1		Patient 2
Age (years)				70		71
Sex				Male		Male
Disease				Lung cancer		Mediastinal tumor (malignant lymphoma)
Surgical procedures				Right lower lobectomy under VATS^a^		Incisional biopsy of a tumor under VATS
Operation time (h: min)				5:06		1:48
Blood loss (mL)				186		11
**Analgesic**						
	Continuous epidural analgia			Fentanyl citrate 0.72 mg mount		None, due to side effect
	Other			Not used		Suppository (2 counts)
**Postoperative course**						
	Resuming walk			POD^b^ 1		POD 1
	Removing the chest drain			POD 2		POD 2
**Measured data**						
	Measurement period			Pre^c^, POD 1-4		Pre, POD 1-3
	**Total number (in whole days)**					
		Measured acceleration data	548,466		466,407	
		All metric data	1,645,398		1,399,221	

^a^VATS: video-assisted thoracoscopic surgery.

^b^POD: postoperative day.

^c^Pre: preoperative day.

### Preprocessing Data

The total number of data points measured for the 2 participants was 1,014,873 (Table 1).

**Figure 1:**
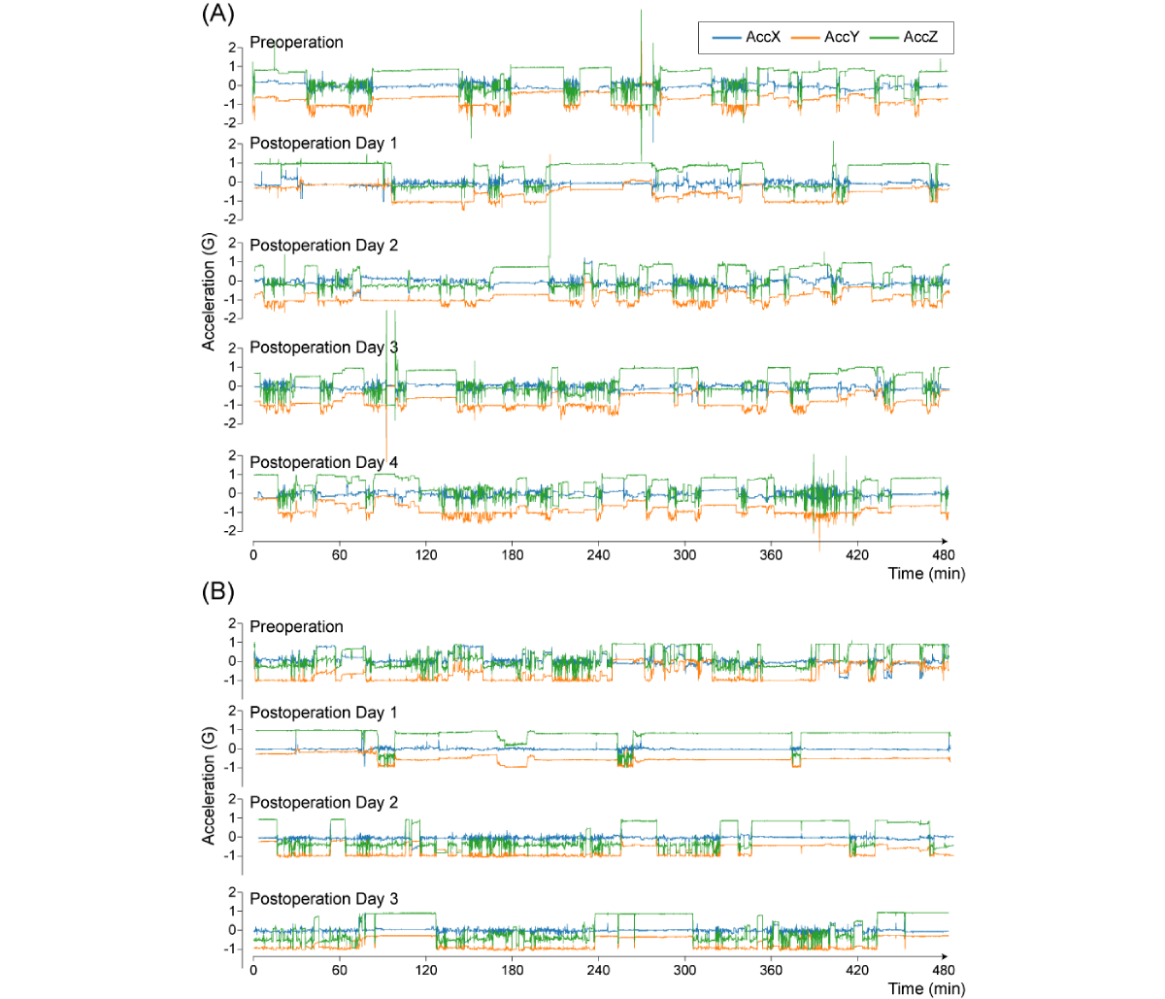
All measured triaxial acceleration data of the patients. (A) Patient 1; (B) Patient 2.

The measured data were recognized as 3 small-amplitude and high-frequency time-series signals with spike noise (Figure 1 provides an overview of the measured data of patient 1).

**Figure 2:**
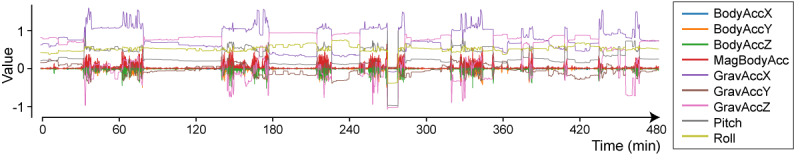
Body and gravitational acceleration data after noise elimination (data from patient 1 on the preoperative day).

BodyAcc and GravAcc were extracted from the measured acceleration data using a high-pass filter with a stopband frequency of 1.5 Hz or a low-pass filter with a passband frequency of 3 Hz. BodyAcc was denoised using a low-pass filter with a stopband frequency of 10 Hz and a Hampel-type filter. GravAcc was denoised using a Hampel-type filter, and the moving average method was used at intervals of 50 (Figure 2 provides an overview of the denoised data of patient 1).

**Figure 3:**
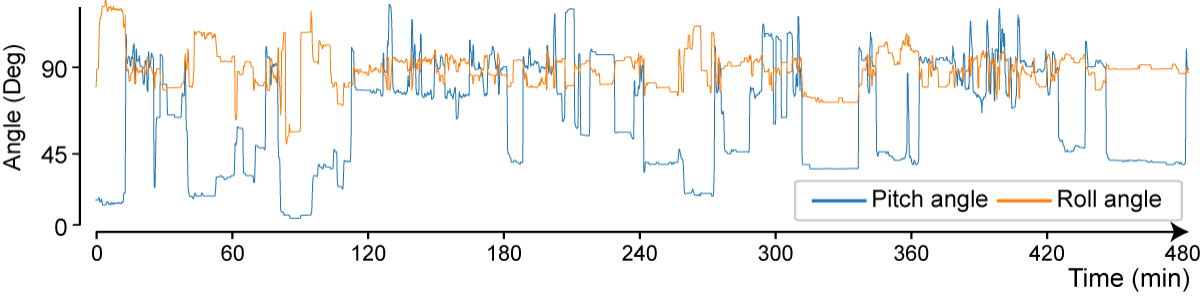
Pitch and roll angles (data from patient 1 on postoperative day 4).

The Euler angles, which are the pitch and roll angles, were calculated from the extracted gravitational acceleration vectors with the direction of gravity as the axis (Figure 3). The pitch angle exhibits a characteristic stepwise waveform that reflects the movement of the upper body.

**Figure 4:**
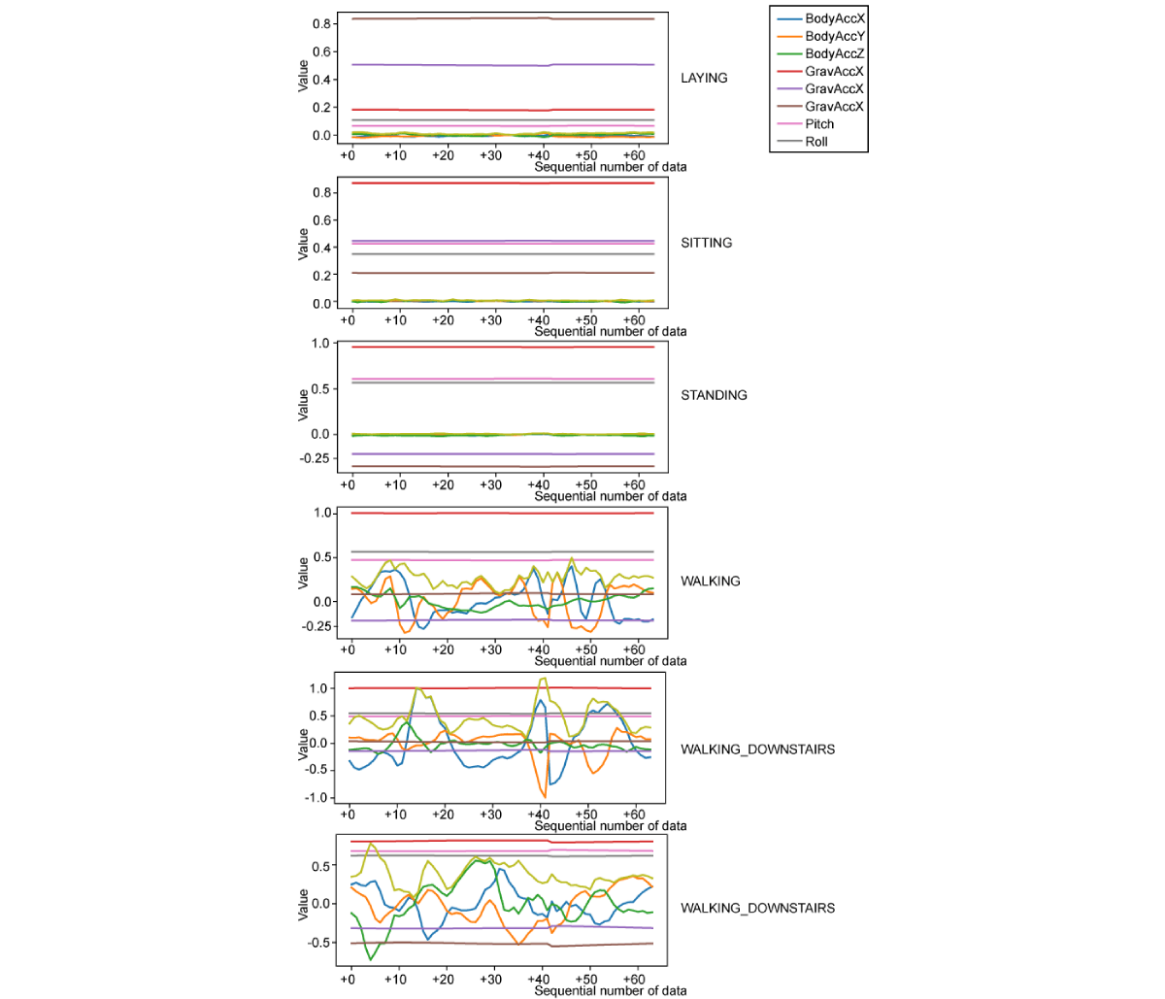
Training data by labels (only part of the supervised data).

The labeled training data showed that 3 WALKING actions (WALKING, WALKING_UPSTAIRS, and WALKING_DOWNSTAIRS) were characterized by periodic changes in acceleration, whereas the acceleration values of the other actions were constant (Figure 4).

### Selecting 3 Learning Models to Predict Actions

**Figure 5:**
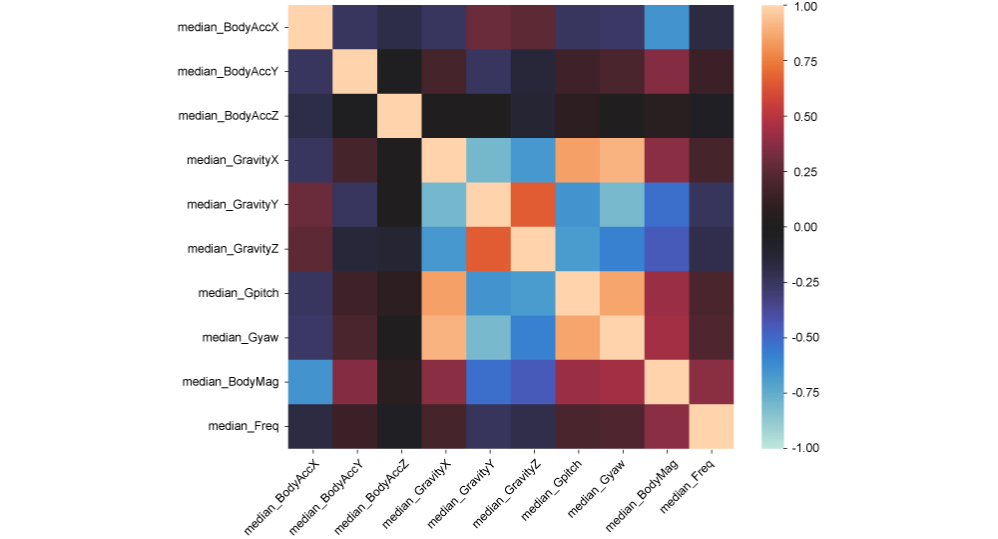
Correlation heat map in terms of median feature data.

**Figure 6:**
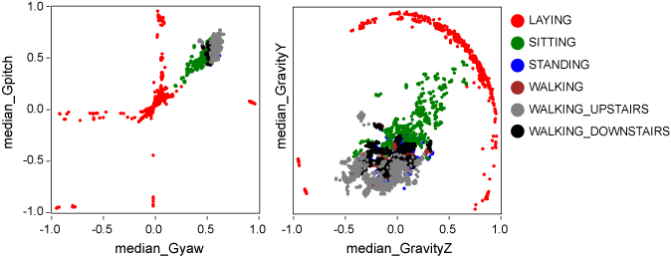
Correlations by label in features with high correlation coefficient.

**Figure 7:**
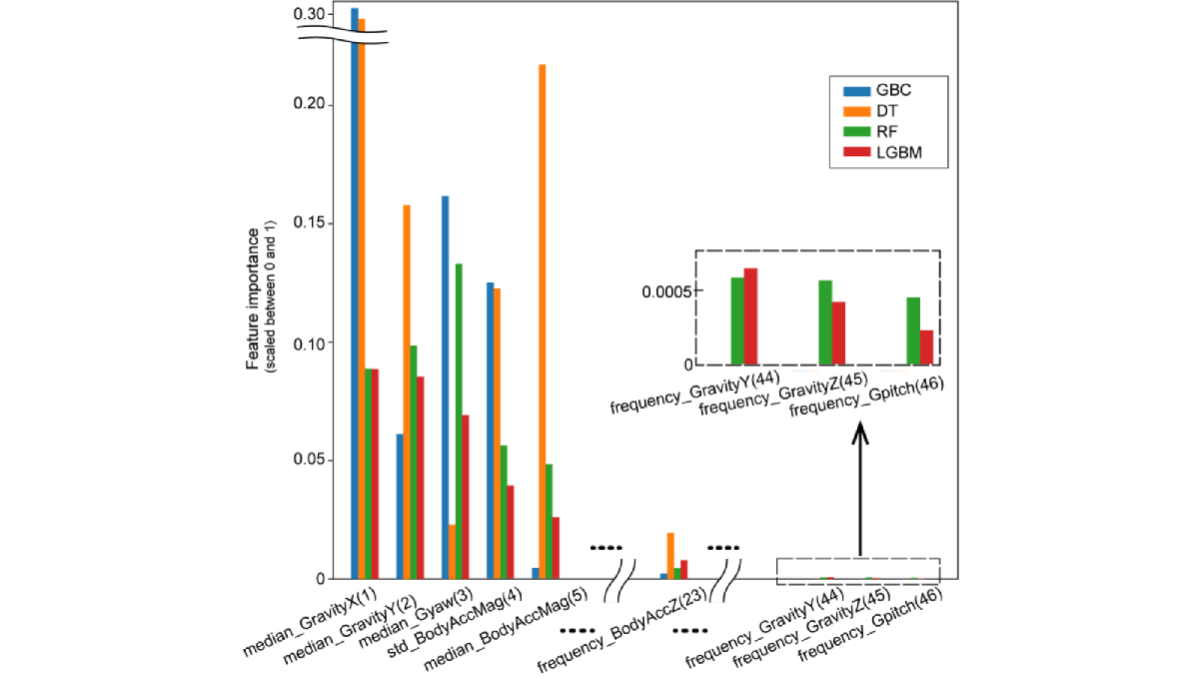
Feature importance. GBC: gradient boosting classifier; DT: decision tree; RF: random forest; LGBM: light gradient boosting method.

In validating the features, the correlation coefficients among the features were high between the median gpitch and yaw and between the “median Gravity Y” and “median Gravity Z” (Figure 5). However, the scatter graph of their correlations shows that they can be used to separate LAYING (Figure 6). Feature importance scores trended to be higher in “median Gravity X” and “median Gravity Y” and lower in features related to the frequency component. However, all features contributed to the prediction (Figure 7).

The cross-validation results for the trained models at the best-tuned parameters showed that the LGBM, GBC, and RF classifiers were the top performers with the highest mean MCCs, and the MCC values and accuracy were LGBM (mean MCC 0.98, SD 0.0027; mean accuracy: 0.98), GBC (mean 0.96, SD 0.0053; accuracy: 0.96), and RF (mean 0.95, SD 0.0079; accuracy: 0.95; Multimedia Appendix 1). The macroaverage accuracy scores for the best MCC scores were as follows: LGBM (MCC: 0.98; accuracy: 0.99; precision: 0.99; recall: 0.99; *F*_1_-score: 0.99), GBC (MCC: 0.97; accuracy: 0.97; precision: 0.97; recall: 0.97; *F*_1_-score: 0.97), and RF (MCC: 0.96; accuracy: 0.96; precision: 0.97; recall: 0.97; *F*_1_-score: 0.97; Multimedia Appendix 2).

**Figure 8:**
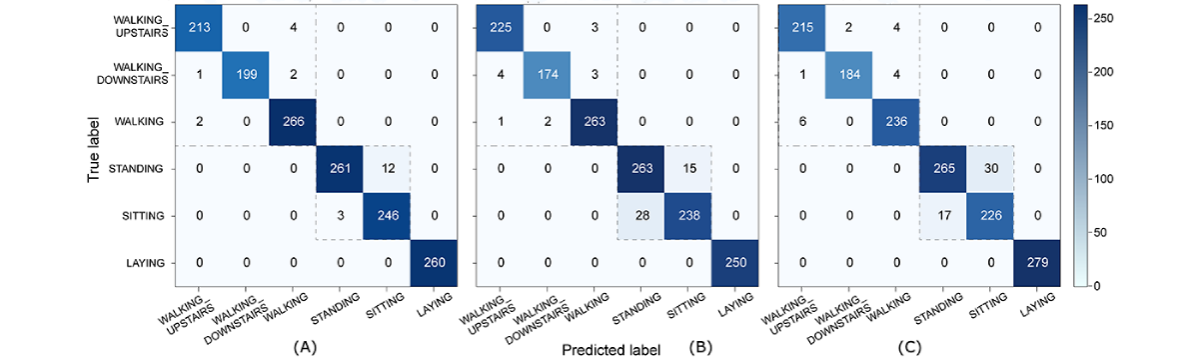
Confusion matrices of the best 3 learned classifiers. (A) Light gradient boosting method; (B) Gradient boosting classifier; (C) Random forest.

An analysis of the confusion matrices of the 3 classifiers showed that all had a 100% accuracy rate for LAYING, with no false positives or false negatives. Few prediction errors were observed between level walking and stair up or down, and most errors were between SITTING and STANDING (Figure 8).

### Aggregation of Predicted Actions

The 3 learned classifiers were provided with the test data and predicted actions. The appearance time per hour was calculated from the results (Multimedia Appendix 3).

**Figure 9:**
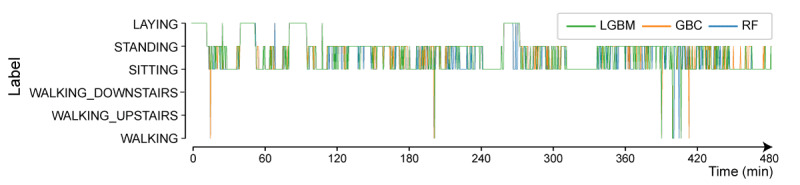
Action time diagram (patient 1, postoperative day 4). LGBM: light gradient boosting method. GBC: gradient boosting classifier. RF: random forest.

Although slight differences in the prediction of the 3 classifiers are observed, they are generally consistent with the overall 8-hour measurement time (Figure 9).

**Figure 10:**
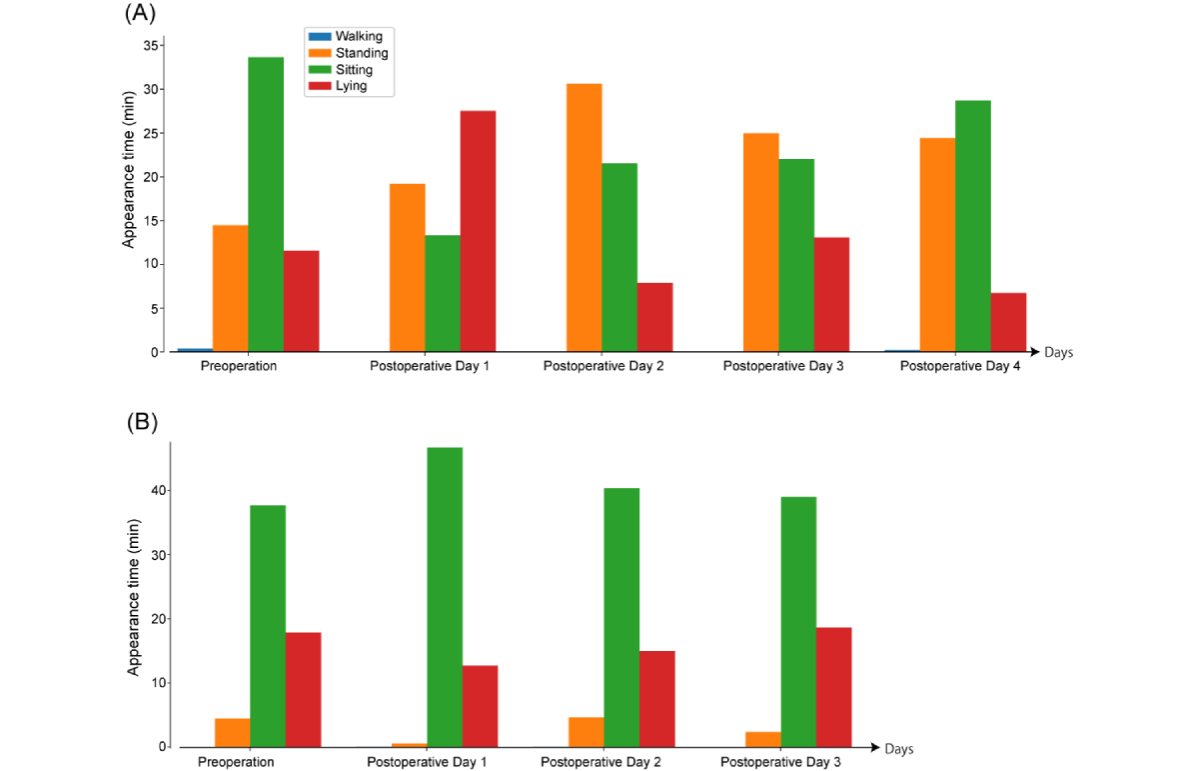
Day-by-day changes in the actions determined by the learned light-gradient boosting method classifier: (A) patient 1 and (B) patient 2.

The LGBM classifier exhibited intermediate values between the other 2 learned classifiers predicting actions using the test data. Therefore, the actions predicted by the LGBM classifier were considered the actions of the patients, and the action frequencies were calculated. Figure 10 shows the day-by-day changes in actions.

Patient 1 spent 11.5 minutes lying down per hour preoperatively and 0.4 minutes walking. On POD 1, the lying time increased to 27.5 minutes, and the walking time was 0.0 minutes. On POD 4, the lying down time decreased to 6.7 minutes, and the walking time increased to 0.2 minutes, which were near the preoperative values. The APly on POD 1 was significantly greater than that on the other days (*P*=.001; 1-sample 1-tailed *t* test).

Patient 2 had a walking time of 0.0 minutes per hour during hospitalization. Their standing time was 4.5 minutes preoperatively, which decreased to 0.5 minutes on POD 1; the lying time decreased from 17.9 to 12.7 minutes. Conversely, their sitting time increased from 37.7 to 44.0 minutes. On POD 2, the percentages of lying down, sitting, and standing times were close to the preoperative values. APsi on POD 1 was greater than that on other days (*P*=.01; 1-sample *t* test). APly on POD 1 was less than the preoperative value; however, the difference was statistically insignificant (*P*=.06; 1-sample *t* test).

The average APst of patient 1 was greater than that of patient 2 (*P*<.001; independent-sample *t* test), whereas APsi of patient 2 was greater than that of patient 1 (*P*=.005; independent-sample *t* test).

### Calculating Behavior Pattern Indices

**Figure 11:**
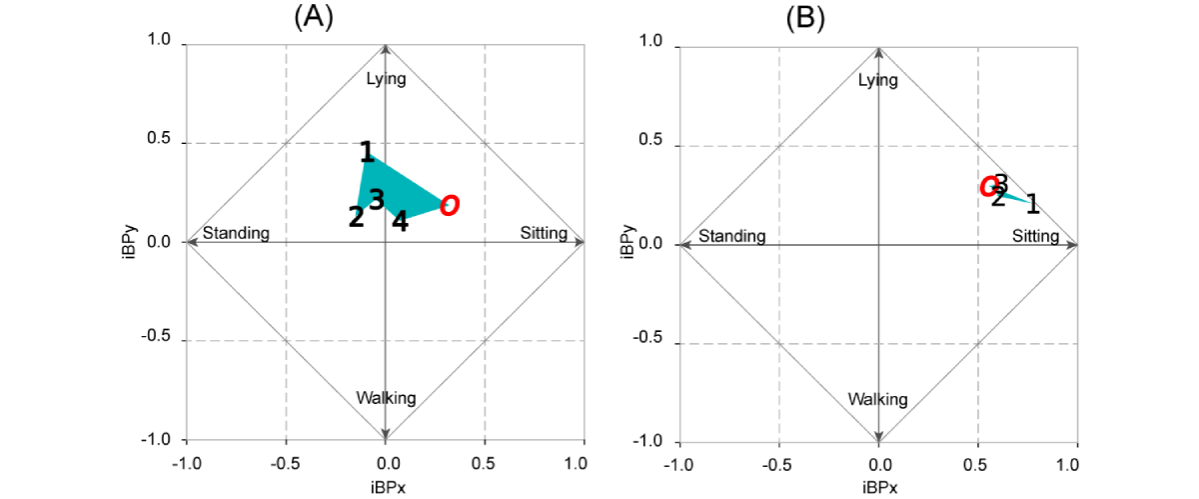
Index of behavior pattern (iBP) on a graph: (A) patient 1 and (B) patient 2.

The iBP values were calculated (Table 2) and plotted to determine the proportional changes for all actions (Figure 11).

In Figure 11, the preoperative index of the behavior pattern is expressed as point O and POD N is expressed as point N on the chart.

**Figure 12:**
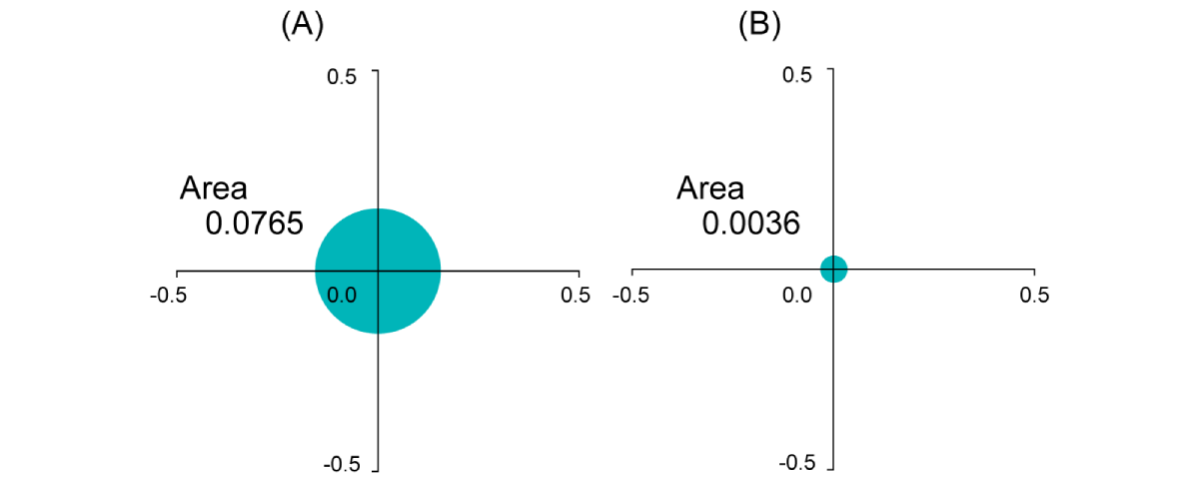
Differences in an area enclosed by behavior pattern indices: (A) patient 1 and (B) patient 2.

The regions enclosed by these points are colored. The area of the region bounded by the shift in coordinates was larger than that of patient 2. Figure 12 shows circles with equal areas enclosed by the iBP points. The differences between the 2 areas became clearer.

The accumulated bar on the left shows the sum of all distances, and the numbers indicate the distances. The bars on the right represent the distance from the preoperative day to the day of the procedure. “to POD N” means from preoperative day to POD N.

The distance gradually decreased after surgery. The values of patient 1 were greater than those of patient 2 (*P*=.03; Mann-Whitney *U* test).

**Figure 13:**
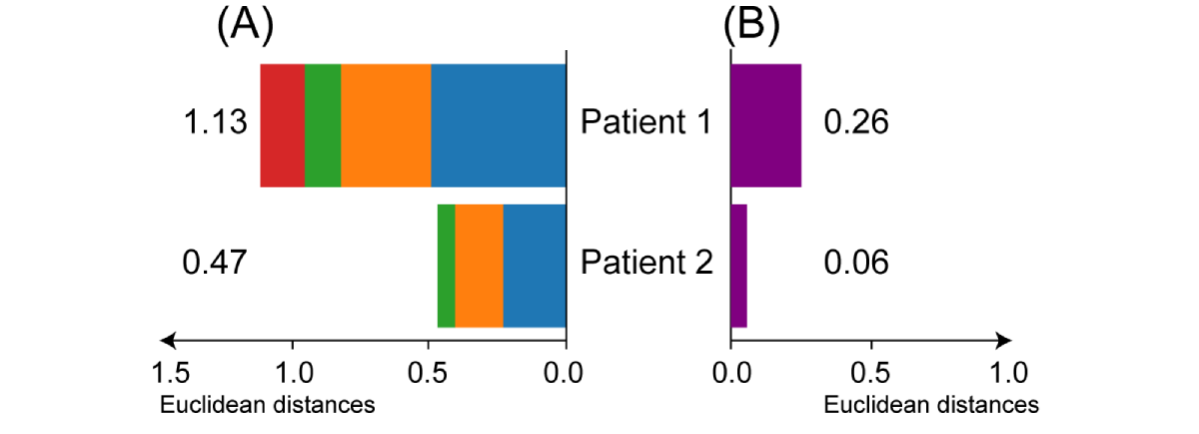
Euclidean distances between behavior pattern indices: (A) total distance and (B) last distance.

Figure 13 illustrates the Euclidean distances between the iBP points on the surgery and preoperative days.

**Table 2:** Change in behavior pattern indices by date.

			Index of behavior patten (x, y)
**Patient 1**			
	Pre^a^	(0.32, 0.19)	
	POD^b^ 1	(−0.098, 0.46)	
	POD 2	(−0.15, 0.13)	
	POD 3	(−0.049, 0.22)	
	POD 4	(0.071, 0.11)	
**Patient 2**			
	Pre	(0.55, 0.30)	
	POD 1	(0.77, 0.21)	
	POD 2	(0.60, 0.25)	
	POD 3	(0.61, 0.31)	

^a^Pre: preoperative day.

^b^POD: postoperative day.

## Discussion

### Principal Findings

The principal findings are summarized as follows: first, by leveraging the latest artificial intelligence technology, the behavior patterns of perioperative patients can be accurately predicted using thoracic movement measurements alone. Second, a novel index derived from changes in behavior patterns demonstrated the potential to quantify surgical invasiveness.

The learned models (decision tree–type estimators) trained with an open data set accurately predicted the behavior actions of a surgical patient. The mean MCCs and accuracies were as follows: LGBM (0.98, 0.98), GBC (0.96, 0.96), and RF (0.95, 0.95). As shown in Figure 10, the time courses of actions predicted by the machine learning model based on the patient’s acceleration data were aligned with the empirically known behavior changes in postoperative patients.

The proposed index, built on each patient’s behavior patterns, effectively visualizes the disparities in physical activity among surgical patients with varying degrees of invasiveness. As shown in Figure 11, in both patients, point 1 (the index of POD 1) was farthest from point O (the preoperative index), and the points gradually moved closer to point O on each day. The differences in movements of the patients’ indices were clearly visualized. The moving distances of patient 1 were significantly greater than those of patient 2 (*P*=.03; Mann-Whitney *U* test).

### Comparison With Prior Work

#### New Perspective on Surgical Invasiveness

**Figure 14:**
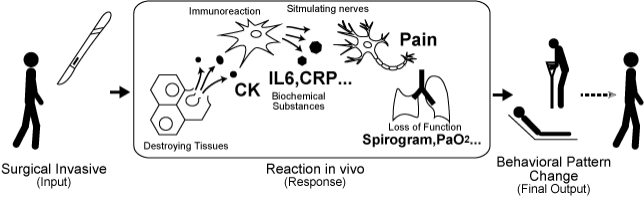
Various indicators of surgical invasiveness. CK: creatine kinase. IL6: interleukin 6. CRP: C-reactive protein. PaO2: partial pressure of arterial oxygen.

Compared with previous studies, a distinctive aspect of this research is the novel approach for assessing surgical invasiveness using the proposed index. This index was designed based on the final response to surgical invasion manifesting as changes in patients’ behavior patterns during the early perioperative period (Figure 14). In addition, this study incorporated the latest advancements in machine learning technology.

#### Patient-Oriented Indicator Independent of Medical Parameters

Unlike conventional indices that rely on chemical or hospital-related parameters, the proposed index adopts a patient-oriented approach. It provides relevant and understandable information to patients, thereby facilitating their transition to daily life. As shown in Figure 11, point 2 of patient 2 has already returned to approximately point O. This indicates that on day 2, patient 2 could act similarly as before the surgery, without an additional load. The primary concern for patients undergoing invasive procedures is the time required to resume their preoperative lifestyle [17] and not the values of chemical or hospital indicators [7,8] unrelated to their daily life activities.

#### Perioperative Indicator Focusing on the Rest State

Although previous studies have often evaluated indicators several weeks after surgery [18-20], this study uniquely assessed the changes in behavior patterns during the early perioperative period. This distinction arises from this study, which focused on the remaining states of surgical patients. Although some prior studies have explored changes in behavior patterns after invasive events, they focused on active levels rather than activity at rest [21,22]. Numerous investigations have aimed to measure postoperative recovery using activity meters and step counts [23-27]; however, these efforts have yielded limited success, particularly during the perioperative period [14]. This might be attributed to the relatively short walking duration observed in hospitalized patients, both before and after the surgery [28-30]. The inherent limitations of the hospital environment, such as confined spaces, can restrict the patients’ ability to walk extensively, diminishing the use of traditional methods to capture subtle variations in their daily activities [31].

We noted a change in the sitting time of patient 2 on POD 1. As expected, the lying time for patient 1 was prolonged after their major surgery. However, the sitting time for patient 2 was prolonged instead of their lying time. This may mean that the level of invasiveness experienced by patient 2 was insufficiently significant to require lying down but did require rest while sitting.

#### Vectorized Index

The proposed index is represented by vector data. Previous studies used simple scalar values [1,7-9,20,32]. Using only scalar indicators, comparisons between days and patients can be executed; vectorization, as illustrated in Figure 11, offers distinct advantages. Although 4D tensorization by the 4 APs is required to retain complete information, this complicates the visualization process.

Therefore, we transformed each scalar AP value into an AP vector by assigning directional values. Subsequently, to unify the 4 *AP* vectors into a single vector, we defined the center-of-gravity vector coordinates based on the 4 *AP* vectors. This vector was established as a new index, iBP.

When using a vectorized representation, the index captures both the direction and magnitude, both of which have clinical significance. By interpreting the preoperative index values as the patient’s origin point, a vector directed toward this origin signified daily recovery. In both patients, the iBP returned was close to its origin, as expected.

The magnitude of the vector, which corresponds to the distance from the origin, reflects the magnitude of the patient’s response to the surgical intervention. Any vector between the 2 indices denotes the magnitude of behavior change during that period, highlighting any increase or decrease in invasiveness.

#### Noninvasive Measurement Method During Perioperative Period

Acceleration was measured noninvasively, devoid of interventions that could impact the patients’ recovery processes, such as exercise testing [33]. The small sensor affixed to the chest wall did not disrupt patient behavior during the perioperative period. This approach allows for painless acceleration measurement and enhances patient comfort and compliance. The sensor was removed at night during sleep to ensure the patient’s ease.

#### Using the HAR Technology Clinical Research Method

HAR technology is a modern approach for monitoring and comprehending human movement and behavior by integrating sensor devices and algorithms, including machine learning [34]. Initially, HAR relied primarily on accelerometers [35], as used in this study. With subsequent advancements in sensor technology, it has evolved to encompass sensor fusion techniques that integrate various sensors, such as gyroscopes, magnetometers, and barometers, and physiological sensors, such as heart rate monitors. This integration enables real-time analyses of complex motions [36]. This technology has already been integrated into numerous smartphones and applied in diverse fields including drone and robot attitude controls [37]. In the medical field, particularly rehabilitation, analyses of walking strides [38] and fall-related events in patients [16] have been reported. The implementation of machine learning continues to emerge in the context of medical research. The HAR technology used in this study represents a fundamental aspect of the current HAR technology. Few studies, such as ours, have applied it as a clinical research method, underscoring its potential and significance [34,35].

### Limitations

#### Validity of the New Index as an Invasiveness Indicator

This study did not assess the validity of the new index as an invasiveness indicator because of the absence of reliable methods for surgical invasiveness [1]. Consequently, we selected patients for 2 cases in which all surgeons agreed on the difference in surgical invasiveness. Although the 2 patients were of the same sex and similar in age, a notable disparity in surgical invasiveness existed between incisional biopsy and radical resection of the lung lobe and lymph nodes. The operation times were 306 and 108 minutes, and the blood loss volumes were 186 and 11 mL, respectively. In future studies, we plan to conduct further research using a larger number of cases.

#### Validity of the Categorization of Actions

We assumed that the breakdown of static position time was essential for evaluating recovery in the early postoperative period. Therefore, this study categorized patient behaviors into 3 static postures (ie, lying, sitting, and standing) and 1 dynamic action (ie, walking). The results sufficiently suggest the patient’s behavior pattern for 8 hours during the daytime, although many other actions and postures are possible.

Current HAR technology cannot completely recognize quick dynamic actions or complex postures without periodicity [39]. Human postural changes are very complex, rapid, and varied to be accurately determined using machine learning. Considering the various actions for behavior prediction would result in poorer accuracy. Fortunately, we determined that walking times in hospitalized patients were short and that patients spent much time at rest. We assumed that other actions and behaviors accounted for a small part of the total action time during hospitalization and could be ignored, such as changes in posture and elevator movements.

#### Validity of the Methodology for Predicting Behavior

Among the many machine learning methods, supervised learning methods are considered more reliable for tasks with statistically strong characteristic data, as in this study [1,13,28,40,41]. As shown in our results, the prediction accuracy was high and reliable. Using the latest artificial intelligence technology enabled us to convert approximately 1 million acceleration data points into a single paired numerical index.

#### Validity of Supervised Data Set

This study used the UPC data set [42] as supervised data because of its reliability. However, most publicly available data sets for HAR [37,43] are problematic because many volunteers supplying their data are young. Supervised data in future studies, particularly in those analyzing dynamic actions, should be collected from older adult volunteers and patients, if possible.

#### Validity of Selection of Measured Items and Features

Although the selected features produced accurate predictions consistent with the objectives of the study, the importance scores for the features associated with the frequency components were lower for the decision tree–type classifier. As a preliminary experiment, multiple iterations were performed using various UPC data set features. Despite these efforts, the prediction accuracy for the 4 action categories did not improve as expected. Consequently, only elementary statistics (median and SD) were selected as features for the triaxial acceleration data. In addition, the amplitude, frequency, and phase values after the fast Fourier transform were selected for detecting walking actions with a wave-like data morphology. Composite acceleration and Euler angles complemented the main items and were expected to reflect the patient’s center-of-gravity acceleration, with a larger mean magnitude indicating more active movement. Among the added features, the median yaw angle of gravity ranked second in the importance scores, just after both the median Gravity X and Y, followed by the SD and median of BodyAccMag (Figure 7). The reason for the overall low importance scores of the frequency components is that the behavior classification in this study focused on static states without considering complex dynamic actions or postural transformations. Therefore, future studies on feature selection are required.

#### Limitation of Detection Using Only a Sensor With Triaxial Acceleration Data

In this study, we selected a sensor that is certified for medical applications in Japan. Using a single sensor presents a challenge in distinguishing between sitting and standing positions. Although the sensor captured only the triaxial acceleration, its prediction accuracy was satisfactory for our study objectives. Fixing the sensor to the anterior chest wall maintains the sensor direction constant and simplifies the behavior prediction. Many public HAR data sets stem from unfixed smartphones that lack direction and location specificity. Another factor that resulted in high accuracy was the surgical patient context. The patients could walk independently throughout the study; however, their behavior was curtailed in the hospital, devoid of running or long walks. We attribute these factors to high prediction accuracy. Although attaching an additional sensor can potentially broaden the range of classified actions, multiple sensors introduce technical complexities such as maintaining a precise synchronization.

### Future Directions

Future studies should validate the proposed index with more cases to determine whether it reflects invasiveness.

Nonetheless, the applicability of this new index is not limited to patients who have undergone surgery. Because it relies solely on patient behavior, it can be extended to evaluate nonsurgically hospitalized patients or individuals requiring nursing care. Daily behavior patterns were encapsulated in an index expressed as a numerical pair between 0 and 1, facilitating statistical comparisons. This inclusivity can extend beyond daily changes within the same patient for comparisons between different patients and procedures.

With the ongoing development of the HAR technology, detailed research on the behavior patterns of patients with more severe conditions or more active patients is possible. Currently, the sensors are equipped with more functions and are smaller than those when this study was designed. The device can detect differences of several centimeters in height based on barometric pressure [44] and track movements using geomagnetic and satellite data. We will be able to grasp the difference between a patient’s sitting and standing positions more easily as well as their speed and range of movement.

We hope that this study inspires medical professionals to conduct medical research using machine learning and to apply the proposed index, conception, and methodology.

### Conclusions

To establish a quantitative measurement method for surgical invasiveness, we focused on behavior patterns in operative patients and proposed an index for the rate of postural time. The index was created using a supervised machine learning method based on the triaxial acceleration data of a patient before and after surgery. The numerical indices clearly demonstrate the difference between 2 patients with different levels of surgical invasiveness on a graph and enable numerical comparisons. The proposed index was created using parameters that do not depend on the type of invasiveness, and we believe that it can be widely applied beyond the evaluation of surgical invasiveness.

## Appendix

Multimedia Appendix 1 Matthews correlation coefficients of the classifiers.

Multimedia Appendix 2 Accuracy classification score among classifiers with the best Matthews correlation coefficients.

Multimedia Appendix 3 The change in the appearance time of the patient’s actions was predicted by the 3 best learned classifiers.

## Abbreviations

AP: appearance probability
GBC: gradient boosting classifier
HAR: human activity recognition
iBP: index of behavior pattern
LGBM: light-gradient boosting method
MCC: Matthews correlation coefficient
POD: postoperative day
RF: random forest
UPC: Universitat Politècnica de Cataluña

## Data Availability

The data are available upon reasonable request from the corresponding author.
