# Supplementary material for: A New Index for the Quantitative Evaluation of Surgical Invasiveness Based on Perioperative Patients’ Behavior Patterns: Machine Learning Approach Using Triaxial Acceleration
Source: JMIR Perioper Med. 2023 Nov 14;6:e50188. doi: 10.2196/50188 (PMC10685283; doi:10.2196/50188)
Supplement: Multimedia Appendix 1 [file periop_v6i1e50188_app1.pdf]

Matthew's correlation coefficient of the classifiers

Classifier	Tuned parameters: the best settings	Mean MCC[a] (Standard Deviation)	Mean Accuracy
LGBM[b]	n[i] leaves: 90, max[j] depth: 10	0.981(0.0027)	0.982
GBC[c]	Learning rate: 0.1, max depth: 3, n estimators: 50	0.958 (0.0053)	0.962
RF[d]	max depth: 4, n estimators: 18	0.945 (0.0079)	0.952
LR[e]	C[k]: 10000	0.896 (0.0128)	0.912
DT[f]	max: depth 9	0.884 (0.0349)	0.904
L-SVC[g]	C: 2, multi_class: crammer_singer	0.871 (0.0313)	0.894
K-SVM[h]	C: 16, Gamma: 0.0078	0.852 (0.0083)	0.878

MCC[a], Matthews Correlation Coefficient; LGBM[b], Light Gradient Boosting Method; GBC[c], Gradient Boosting Classifier; RF[d], Random Forest; LR[e], Logistic Regression; DT[f], Decision Tree; L-SVC[g], Linear type Support Vector Classifier; K-SVM[h], Kernel type Support Vector Method; n[i], number of; max[j], maximum; C[k],C parameter
