# Supplementary material for: A New Index for the Quantitative Evaluation of Surgical Invasiveness Based on Perioperative Patients’ Behavior Patterns: Machine Learning Approach Using Triaxial Acceleration
Source: JMIR Perioper Med. 2023 Nov 14;6:e50188. doi: 10.2196/50188 (PMC10685283; doi:10.2196/50188)
Supplement: Multimedia Appendix 2 [file periop_v6i1e50188_app2.pdf]

Accuracy classification score among the classifiers with the best Matthews correlation coefficient

Classifier (best MCC [a])	Accuracy	Average type	Precision	Recall	F1-score
LGBM[b]	0.99	M[i]	0.99	0.99	0.99
(0.98)		W[j]	0.99	0.99	0.99
GBC[c]	0.97	M	0.97	0.97	0.97
(0.97)		W	0.97	0.97	0.97
RF[d]	0.96	M	0.97	0.97	0.97
(0.96)		W	0.96	0.96	0.96
LR[e]	0.93	M	0.93	0.93	0.93
(0.92)		W	0.93	0.93	0.93
DT[f]	0.94	M	0.93	0.93	0.93
(0.92)		W	0.94	0.94	0.94
L-SVC[g]	0.91	M	0.90	0.90	0.90
(0.89)		W	0.91	0.91	0.91
K-SVM[h]	0.89	M	0.89	0.88	0.89
(0.86)		W	0.89	0.89	0.89

MCC[a], Matthews Correlation Coefficient; LGBM[b], Light Gradient Boosting Method; GBC[c], Gradient Boosting Classifier; RF[d], Random Forest; LR[e], Logistic Regression; DT[f], Decision Tree; L-SVC[g], Linear type Support Vector Classifier; K-SVM[h], Kernel type Support Vector Method; M[i], macro average; W[j], weighted average
