# Supplementary material for: A New Index for the Quantitative Evaluation of Surgical Invasiveness Based on Perioperative Patients’ Behavior Patterns: Machine Learning Approach Using Triaxial Acceleration
Source: JMIR Perioper Med. 2023 Nov 14;6:e50188. doi: 10.2196/50188 (PMC10685283; doi:10.2196/50188)
Supplement: Multimedia Appendix 3 [file periop_v6i1e50188_app3.pdf]

Change in the appearance time of the subject's actions predicted by the best three learned classifiers

Classifier	Label	Appearance Time per hour[min]								
		Patient 1					Patient 2			
		Pre[a]	POD[b]				Pre	POD		
			1	2	3	4		1	2	3
GBC[c]	Walking All	0.2	0	0.1	0	0.3	0	0	0	0
	-WLK[d]	0.2	0	0.1	0	0.2	0	0	0	0
	-WUS[e]	0	0	0	0	0.1	0	0	0	0
	-WDS[f]	0	0	0	0	0.1	0	0	0	0
	SIT[g]	30.7	9.5	15.4	15.4	22.3	32.1	41.5	30.8	32.4
	STD[h]	19.9	23.5	40.3	30.4	30.8	9.7	5.6	11.7	6.5
	LAY[i]	19.2	27	4.2	14.1	6.7	18.1	12.9	17.5	21.2
LGBM[j]	Walking All	0.4	0	0	0	0.2	0	0.05	0.05	0
	-WLK	0.3	0	0	0	0.2	0	0.05	0.05	0
	-WUS	0.1	0	0	0	0	0	0	0	0
	-WDS	0	0	0	0	0	0	0	0	0
	SIT	33.6	13.3	21.5	22	28.7	37.7	46.7	40.4	39
	STD	14.5	19.2	30.6	25	24.4	4.5	0.5	4.6	2.3
	LAY	11.5	27.5	7.9	13.1	6.7	17.9	12.7	15	18.7
RF[k]	Walking All	0.4	0	0.1	0	0.2	0	0	0	0
	-WLK	0.4	0	0.1	0	0.2	0	0	0	0
	-WUS	0	0	0	0	0.1	0	0	0	0
	-WDS	0	0	0	0	0	0	0	0	0
	SIT	35	15.9	28.7	22.7	31.4	35.8	44	37.5	35.3
	STD	12.8	14.1	23.5	22.8	21	3.7	1.3	4.8	3.8
	LAY	11.9	30	7.8	14.4	7.4	20.5	14.6	17.6	20.9

Pre[a], preoperative day; POD[b], postoperative day; GBC[c], Gradient Boosting Classifier; WLK[d],WALKING; WUS[e], WALKING_UPSTAIRS; WDS[f],

WALKING_DOWNSTAIRS; SIT[g], SITTING; STD[h], STANDING; LAY[i,] LAYING; LGBM[d], Light Gradient Boosting Method; RF[k], Random Forest
